# Complete Genome Sequence of *Pseudomonas aeruginosa* Phage vB_PaeM_CEB_DP1

**DOI:** 10.1128/genomeA.00918-15

**Published:** 2015-09-24

**Authors:** Diana P. Pires, Sanna Sillankorva, Andrew M. Kropinski, Timothy K. Lu, Joana Azeredo

**Affiliations:** a Centre of Biological Engineering, University of Minho, Braga, Portugal; b Public Health Agency of Canada, Laboratory for Foodborne Zoonoses, Guelph, Ontario, Canada; c Department of Molecular and Cellular Biology, University of Guelph, Guelph, Ontario, Canada; d Department of Electrical Engineering and Computer Science, Massachusetts Institute of Technology, Cambridge, Massachusetts, USA; e Department of Biological Engineering, Massachusetts Institute of Technology, Cambridge, Massachusetts, USA

## Abstract

vB_PaeM_CEB_DP1 is a *Pseudomonas aeruginosa* bacteriophage (phage) belonging to the *Pbunalikevirus* genus of the *Myoviridae* family of phages. It was isolated from hospital sewage. vB_PaeM_CEB_DP1 is a double-stranded DNA (dsDNA) phage, with a genome of 66,158 bp, containing 89 predicted open reading frames.

## GENOME ANNOUNCEMENT

The lytic bacteriophage vB_PaeM_CEB_DP1 was isolated from hospital sewage in Portugal using *Pseudomonas aeruginosa* PAO1 as the host strain. Its host range was evaluated using a panel of 30 *P. aeruginosa* clinical isolates, and this phage was able to infect approximately 57% of them.

The morphological characterization of phage vB_PaeM_CEB_DP1 was performed by transmission electron microscopy, revealing an icosahedral head of ~70 nm in diameter and an ~140- × 18-nm contractile tail. Thus, it was possible to determine that this phage belongs to the *Myoviridae* family of phages. Furthermore, the growth parameters determined by the one-step growth experiment showed that phage vB_PaeM_CEB_DP1 has a latent period of ~50 min, a rise period of ~50 min, and a burst size of ~70 phages per infected cell.

The phage genome was sequenced using Roche 454 sequencing procedures at the Plateforme d’analyses of the Institut de Biologie Intégrative et des Systèmes (Laval University, Québec, QC, Canada). Shotgun reads were assembled using the gsAssembler module of Newbler v 2.5.3.

The potential coding sequences (CDSs) were first annotated using myRAST (1). Sequence similarity searches were performed with the translation of each predicted CDS against the National Center for Biotechnology Information (NCBI) protein database, using BLASTP (2), in order to assign putative protein functions. Promoter sequences were predicted based on their similarity to promoter sequences from other *Pbunalike* phages (3). Predicted terminators were annotated using ARNold (4). The tool tRNAscan-SE (5) was used for tRNA annotation but, similarly to other *Pbunalike* phages (3, 6, 7), no putative genes coding for tRNAs were found in this genome.

The genome of the phage vB_PaeM_CEB_DP1 consists of 66,158 bp of dsDNA with a GC content of 55.6%. The whole genome was scanned for CDSs, resulting in 89 predicted genes ranging from 141 bp to 3,111 bp. Furthermore, 37 of these genes are rightward oriented while 52 are leftward oriented. The initiation codon of 90% of the genes is ATG, while 8% start with GTG and 2% with TTG. According to BLASTP analyses, 68% of the proteins encoded in the genome of vB_PaeM_CEB_DP1 are hypothetical. This study further revealed that this phage has 7 predicted promoters and 12 terminators.

Although controversial, most of the phages belonging to the *Pbunalike* genus are reported to encode linear, nonpermuted genomes (3, 6). In the present study, direct Sanger sequencing of phage DNA was performed to determine the genome ends of phage vB_PaeM_CEB_DP1. However, the ends of the genome were not identified, suggesting that the phage genome has cohesive ends or terminal redundancy as described for phage KPP12 (7).

The genome of phage vB_PaeM_CEB_DP1 shares high nucleotide identity with other *P. aeruginosa Pbunalike* phages: LMA2 (95.6%), KPP12 (94.3%) and vB_PaeM_PAO1_Ab27 (93.1%).

### Nucleotide sequence accession number.

The complete genome of the *P. aeruginosa* phage vB_PaeM_CEB_DP1 was deposited in GenBank under the accession number KR869157.
